# FpFumB Is Required for Basic Biological Processes and Virulence in *Fusarium proliferatum* by Modulating DNA Repair Through Interaction with FpSae2

**DOI:** 10.3390/microorganisms13061433

**Published:** 2025-06-19

**Authors:** Yizhou Gao, Haibo Li, Yong Liu, Yuqing Wang, Jingwen Xue, Yitong Wang, Zhihong Wu

**Affiliations:** School of Biological and Chemical Engineering, Zhejiang University of Science and Technology, Hangzhou 310023, China

**Keywords:** *Fusarium proliferatum*, fumarase, TCA cycle, pathogenicity, stress response, DNA damage repair

## Abstract

Fumarase plays a pivotal role in the tricarboxylic acid cycle, but its functions in plant pathogenic fungi are not well understood. We identified two fumarase genes in *Fusarium proliferatum* and generated individual deletion mutants. Loss of *FpFumB* led to defects in growth, sporulation, stress tolerance, and virulence. Exogenous malate supplementation restored growth defects. Site-directed mutagenesis of residues G452 and A463 reduced FpFumB enzyme activity. Transcriptomic analysis identified significant changes in gene expression related to different metabolic pathways. Protein interaction assays showed that FpFumB interacts with the DNA repair protein FpSae2. Both Δ*FpFumB* and Δ*FpSae2* mutants displayed altered sensitivity to DNA-damaging agents and reduced virulence, indicating that FpFumB modulates DNA repair and pathogenicity through its interaction with FpSae2. Together, these findings highlight FpFumB as a key regulator of basic biological processes, DNA damage repair, and virulence in *Fusarium proliferatum*.

## 1. Introduction

*Fusarium proliferatum* is a filamentous fungal pathogen of considerable economic and agricultural significance due to its broad host range and ability to synthesize harmful mycotoxins [1]. It infects a wide variety of crops, including maize, sorghum, and rice, as well as ornamentals such as orchids, resulting in both quantitative yield losses and qualitative degradation of produce [2]. One of the most notable threats posed by *F. proliferatum* is its production of fumonisins and moniliformin—mycotoxins associated with serious health risks in humans and animals [3,4]. The molecular basis of *F. proliferatum* pathogenicity involves the expression of key virulence genes, the production of toxic secondary metabolites, and dynamic responses to environmental cues. In addition to secreting cell-wall-degrading enzymes (CWDEs) and generating reactive oxygen species (ROS), the fungus also produces volatile organic compounds (VOCs), which are recognized as key mediators of host–pathogen interactions [5,6,7]. On the molecular level, multiple genes associated with pathogenicity have been functionally characterized. For instance, *FpFumB1* serves as a key regulator within the fumonisin biosynthesis gene cluster [8,9], *FpOGT* encodes an O-GlcNAc transferase that influences both metabolic flux and signal transduction [10], *FpOPSB* likely contributes to host tissue colonization through its role as a secreted aspartic protease [11], and *FpFPK1*, a mitogen-activated protein kinase, has been implicated in environmental signal perception and stress response regulation [12]. Despite recent progress, the molecular mechanisms underlying *F. proliferatum* pathogenicity remain incompletely understood.

Fumarase (also known as fumarate hydratase) is an important metabolic enzyme that catalyzes the reversible conversion of fumarate to L-malate in the tricarboxylic acid (TCA) cycle [13]. This reaction is essential for energy production in cells and helps maintain the balance of redox molecules like NADH and NAD^+^ [14]. Fumarase is mainly found in mitochondria, where it supports ATP synthesis and normal respiration [15]. However, research shows that fumarase can also be found in the cytosol and nucleus. This means it may have other roles besides metabolism [16]. Its presence in different parts of the cell is related to changes in how it is made, modified after translation, or transported from the mitochondria [17]. These findings suggest that fumarase may perform different functions depending on where it is located in the cell.

Emerging studies have revealed that its biological functions extend far beyond core metabolism, with crucial physiological roles in both plants and microorganisms. In *Arabidopsis thaliana*, two isoforms of fumarase have been identified, one localized to the mitochondria and the other in the cytosol. The cytosolic isoform plays a critical role in the accumulation of fumarate derived from photosynthetic carbon, especially under high nitrogen supply [18]. Plants lacking cytosolic fumarase exhibit drastically reduced fumarate levels and show significantly slower growth, indicating its importance in carbon partitioning and biomass production [19]. Moreover, the same mutant lines display increased sensitivity to both high and low temperature stress, suggesting that cytosolic fumarase also contributes to thermal acclimation through metabolic buffering [19]. Biochemical studies have shown that although the mitochondrial AtFUM1 and cytosolic AtFUM2 isoforms share similar kinetic properties, they differ significantly in redox sensitivity, pH response, and allosteric regulation, allowing plants to modulate carbon flow direction in response to environmental and metabolic cues [20]. In *Solanum lycopersicum*, suppression of mitochondrial FH activity results in impaired root development and altered stomatal conductance, leading to reduced photosynthetic performance and morphological changes in the root system [21]. In *Escherichia coli*, a triple mutant (Δ*fumACB*) completely lacking fumarase is unable to respire and therefore unable to grow on minimal media with acetate as a sole carbon and energy source. Additionally, in the absence of FumC, FumA and FumB can participate in respiration but likely play a less critical role in this function [22]. In *Pseudomonas aeruginosa*, fumarase (FumC) is crucial for metabolic adaptation to iron deprivation, displaying heightened activity that supports increased alginate production [23]. These findings highlight fumarase as a multifaceted enzyme involved not only in energy metabolism but also in stress response and metabolite regulation across plant and microbial systems.

Fumarase, primarily recognized for its role in the TCA cycle, has been increasingly linked to DNA damage repair mechanisms [24]. In *Saccharomyces cerevisiae*, fumarase is crucial for the homologous recombination repair pathway, where it facilitates the recruitment of Sae2 to double-strand breaks, promoting effective resection and repair [25]. Sae2 is a conserved eukaryotic protein that aids in the early processing of DNA double-strand breaks by promoting end resection and bridging [26]. It works with the Mre11-Rad50-Xrs2 complex to initiate homologous recombination repair by creating 3’ single-stranded DNA overhangs [26]. Research indicates that Sae2 activity is influenced by the metabolic enzyme fumarase, linking cellular metabolism to DNA repair efficiency [27]. Furthermore, studies have shown that fumarase localization to the nucleus is essential for its function in DNA repair, suggesting a direct involvement in managing genomic stability [13,28]. In *Escherichia coli*, fumarase influences the repair of oxidative DNA damage by modulating the expression of repair proteins, thereby enhancing cellular resilience to genotoxic stress [29,30]. Additionally, it was demonstrated that fumarase from *Bacillus subtilis* is induced upon DNA damage, co-localizes with bacterial DNA, and is essential for the DNA damage response, with its activity linked to the production of L-malic acid, which modulates the translation of RecN, a key protein in the DNA repair process [31]. These specific findings affirm the significant role of fumarase in DNA repair across various microbial systems, indicating that it plays a protective role against DNA damage by interacting with key repair pathways and proteins.

While fumarase has been extensively studied in model organisms for its roles in energy metabolism and genome maintenance, its biological functions in phytopathogenic fungi remain largely unknown. In this study, we identified and functionally analyzed two fumarase-encoding genes, *FpFumA* and *FpFumB*, in *F. proliferatum*. Through gene knockout, phenotypic assays, metabolic profiling, transcriptomics, and protein interaction studies, we provide comprehensive insights into the roles of these genes in fungal development, environmental stress response, virulence, mitochondrial metabolism, and DNA repair. Our findings reveal that FpFumB, in particular, serves as a pivotal regulator of fungal physiology and pathogenicity.

## 2. Materials and Methods

### 2.1. Fungal Strains and Culture Conditions

The *F. proliferatum* strain HM19-1-1 was used as the wild-type (WT) strain for generating gene deletion mutants and conducting fungal transformation experiments [32]. All fungal strains were cultured on potato dextrose agar (PDA) plates at 26 °C to observe general growth and colony morphology. For sporulation analysis, fungal cultures were transferred to carboxymethyl cellulose (CMC) liquid medium and incubated at 28 °C with constant shaking at 200 rpm for 24 or 48 h. The conidia were collected and counted using a hemocytometer, and their morphological characteristics were observed under a microscope. For each sample, 100 conidia were measured, and their lengths were determined using ImageJ 1.8.0 software. To test stress tolerance, fungal cultures were grown on PDA plates containing Congo Red (CR) (0.2%) (Sigma-Aldrich, St. Louis, MO, USA), Calcofluor White (CFW) (0.2%) (Sigma-Aldrich, St. Louis, MO, USA), sodium dodecyl sulfate (SDS) (0.05%) (Sigma-Aldrich, St. Louis, MO, USA), NaCl (2 mol/L) (Sigma-Aldrich, St. Louis, MI, USA), CaCl_2_ (1.4 mol/L) (Sigma-Aldrich, St. Louis, MO, USA), HU (hydroxyurea) (10 mmol/L) (Sigma-Aldrich, St. Louis, MO, USA), MMS (methyl methanesulfonate) (30 mmol/L) (Sigma-Aldrich, St. Louis, MO, USA), or H_2_O_2_ (2 mmol/L) (Sigma-Aldrich, St. Louis, MI, USA). The growth inhibition rate was calculated as [(control colony diameter − treated colony diameter)/control colony diameter] × 100%.

### 2.2. Gene Deletion and Complementation Constructs

Gene deletion mutants were constructed using a double-joint PCR technique, which involved the amplification of the upstream and downstream homologous arms flanking the *FpFUM* gene, followed by fusion with a hygromycin resistance cassette [33]. The fusion product was introduced into *F. proliferatum* protoplasts via PEG-mediated transformation, and transformants were selected on PDA plates containing 100 mg/mL hygromycin B. Selected transformants were confirmed by PCR and qPCR (quantitative PCR) to analyze the integration of the cassette into the fungal genome. For the complementation experiments, the *FpFUM* gene, including its promoter region, was cloned into the pYF11 vector, which also contained a G418 resistance gene fragment. This construct was transformed into the respective gene deletion mutants, and transformants were selected on PDA plates containing 50 µg/mL G418. For the construction of the Δ*FpFumB*::FpSae2 overexpression strain, the *FpSae2* gene was cloned into the pYF11 expression vector containing a GFP tag, and the resulting plasmid was transformed into the Δ*FpFumB* mutant. Successful transformants were selected on PDA medium containing G418 and then verified by PCR.

### 2.3. Virulence Assays in Medicago Sativa

To assess the virulence of the fungal strains, a seed infection assay was performed using *Medicago sativa*. Sterilized seeds were placed on water agar (WA) plates inoculated with fungal strains and incubated at 28 °C for 10 days. Disease severity was assessed using a 0–4 grading scale, with Grade 0 representing healthy seedlings and Grade 4 indicating severe seedling rot. The disease index was calculated based on these severity ratings from three biological replicates.

### 2.4. RNA Extraction and Quantitative PCR

RNA was isolated from fungal mycelia using the RNA isolator reagent (Vazyme, Nanjing, China), following the manufacturer’s protocol. cDNA synthesis was carried out using the HiScript III First Strand cDNA Synthesis Kit (Vazyme, Nanjing, China). The cDNA was then used for qPCR using ChamQ SYBR qPCR Master Mix (Vazyme, Nanjing, China) on an Applied Biosystems 7500 system. The relative gene expression was quantified using the 2^−ΔΔCT^ method, with *FpActin* (FPRO_04359) as the internal reference gene. Primer sequences are listed in Table S1.

### 2.5. RNA Sequencing

RNA sequencing was performed to compare gene expression between the WT and Δ*FpFumB* strains. Mycelia were collected after 3 days of growth in potato dextrose broth (PDB) at 28 °C and immediately frozen in liquid nitrogen. RNA was extracted, and library construction was performed by Applied Protein Technology (Shanghai, China). The resulting RNA libraries were sequenced using Illumina technology. The quality of raw data was assessed using FastQC v0.11.4, and clean reads were aligned to the *F. proliferatum* ET1 reference genome using TopHat2 v2.1.0 [34]. Differential gene expression analysis was conducted with DESeq2 v1.26.0, and functional enrichment was performed using Gene Ontology (GO) and KEGG (Kyoto Encyclopedia of Genes and Genomes) pathway analyses.

### 2.6. Western Blot

Mycelia were harvested and lysed in RIPA buffer (Beyotime, Shanghai, China) supplemented with protease inhibitors, and protein concentrations were determined using the BCA assay. Proteins were separated by SDS-PAGE, transferred to a PVDF membrane, and blocked for 1 h at room temperature. The membrane was incubated overnight with primary antibodies and then incubated with HRP-conjugated secondary antibodies. Protein bands were detected using the Immobilon Western Chemiluminescent HRP Substrate (Merck Millipore, Darmstadt, Germany), and images were captured with the Omega Lum C imaging system (Aplegen, San Francisco, CA, USA), following the procedure described previously [35].

### 2.7. Yeast Two-Hybrid Assay

The *FpSae2* gene was cloned into the pGBKT7 vector, which contains the GAL4 DNA-binding domain, and *FpFumB* was cloned into the pGADT7 vector, which carries the GAL4 activation domain. The plasmid constructs were verified by DNA sequencing and co-transformed into *Saccharomyces cerevisiae* strain Y2H Gold using the lithium acetate method. The transformed yeast cells were plated on selective medium DDO (SD/-Trp/-Leu) (Clontech, Mountain View, CA, USA) to select for successful co-transformation. The protein interaction was assessed by plating the yeast cells on QDO (SD/-Trp/-Leu/-His/-Ade) (Clontech, Mountain View, CA, USA). As a positive control, the known interacting pGBKT7-53 and pGADT7-T constructs were used as previously described [36].

### 2.8. Co-Immunoprecipitation Assay

To generate strains for the Co-Immunoprecipitation (Co-IP) assay, the *FpSae2* gene was cloned into the pYF11 vector containing a GFP tag, and the *FpFumB* gene was cloned into the pHZ126 vector containing a Flag tag. Both constructs were then co-transformed into the WT strain. Successful transformants were selected on PDA medium containing both hygromycin (Roche Diagnostics, Mannheim, Germany) and G418 (Thermo Fisher Scientific, Waltham, MA, USA), and then further verified by PCR. Fungal mycelia were ground in liquid nitrogen, and proteins were extracted using Co-IP buffer with protease inhibitors. Lysates were incubated with anti-GFP antibodies (Abcam, Cambridge, UK), and the immunocomplexes were precipitated using protein A/G beads (ChromoTek, PlaneggMartinsried, Germany). The precipitates were analyzed by Western blotting to identify co-precipitated proteins, confirming interactions between FpFumB and FpSae2. The methods and operations were conducted as previously described [37].

### 2.9. Mitochondrial Function and Metabolic Assays

Fungal mycelia were harvested and ground in liquid nitrogen, followed by centrifugation to obtain the supernatant. Fumarase activity was determined by incubating 20 µL of enzyme extract with 180 µL of reagent and measuring the absorbance at 240 nm. H_2_O_2_ content was measured using a Hydrogen Peroxide Detection Kit (Beyotime, Shanghai, China), where 50 µL of the supernatant was incubated with 100 µL of reagent, and absorbance was read at 560 nm. ATP levels were quantified by mixing 20 µL of supernatant with 100 µL of ATP detection reagent (Beyotime, Shanghai, China), and the emitted light was measured in relative light units (RLUs) using a chemiluminescence reader.

### 2.10. Protein Prokaryotic Expression

Site-directed mutagenesis of FpFumB was performed using the Mut Express Universal Fast Mutagenesis Kit (Vazyme, Nanjing, China) to generate the *FpFumB*^R39A^, *FpFumB*^V371I^, *FpFumB*^G452A^, and *FpFumB*^A463Q^ mutants. The mutated genes were then verified by DNA sequencing. These mutant genes were then cloned into the pET32a prokaryotic expression vector containing the His-tag. The recombinant protein was expressed in *E. coli* by inoculating 200 µL of overnight culture into 100 mL LB medium containing the appropriate antibiotic. The culture was grown to an OD600 of 0.6–0.8, and protein expression was induced by adding 1 mM IPTG (Isopropyl β-D-1-thiogalactopyranoside) (Sigma-Aldrich, St. Louis, MI, USA) and incubating at 28 °C for 6 h. After induction, the cells were harvested by centrifugation and resuspended in PBS (phosphate-buffered saline) (Beyotime, Shanghai, China) containing DTT (dithiothreitol) (Beyotime, Shanghai, China), PMSF (phenylmethylsulfonyl fluoride) (Beyotime, Shanghai, China), and a protease inhibitor cocktail. Cell lysis was achieved by sonication, followed by centrifugation to collect the supernatant, which was stored for purification. Protein purification was performed using affinity chromatography with an antibody-conjugated resin.

## 3. Results

### 3.1. Regulation of FpFUM Genes on Basic Biological Processes of Fusarium proliferatum

**Figure 1:**
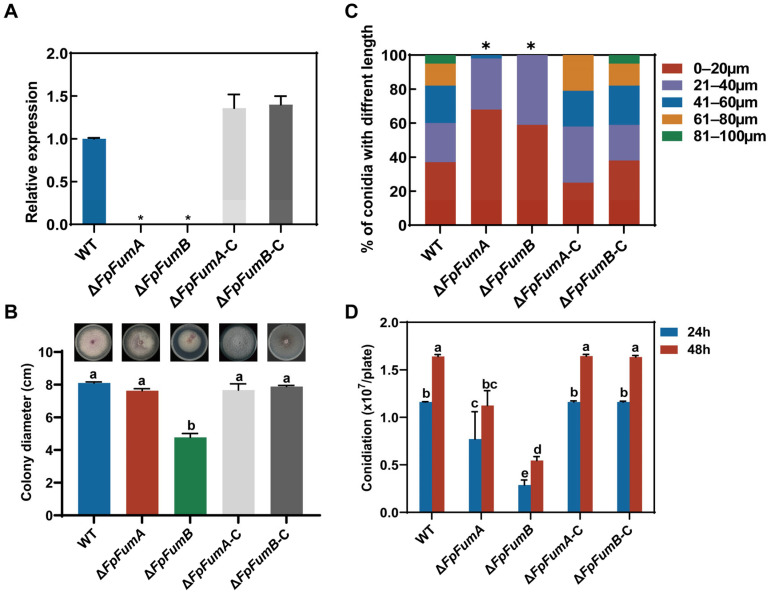
Functional analysis of FpFumA and FpFumB in *Fusarium proliferatum* growth, conidial development, and sporulation. (**A**) Relative expression levels of *FpFumA* and *FpFumB* in wild-type (WT), Δ*FpFumA*, Δ*FpFumB*, and complemented strains, validated by qPCR. Data are normalized to WT expression levels (set as 1.0). (**B**) Colony diameter comparisons of WT and mutants after 7-day growth on PDA medium, visualized with representative colony images. (**C**) Percentage distribution of conidial lengths (0–20 μm, 21–40 μm, 41–60 μm, 61–80 μm, 81–100 μm) in WT and mutants. (**D**) Conidiation levels of WT and mutants at 24 h and 48 h. Error bars represent standard deviation. Statistical significance markers (*) and lowercase letters denote between-group comparisons (ANOVA with post hoc analysis).

Through homologous sequence alignment and protein domain analysis, we identified two fumarase genes in the *F. proliferatum* genome, namely *FpFumA1* (FPRO_09626, XM_031233037) and *FpFumA2* (FPRO_06773, XM_031228277). To investigate the biological functions of the *FpFUM* genes, we constructed knockout mutants of these genes using a homologous recombination strategy (Figure S1A), resulting in the Δ*FpFumA* and Δ*FpFumB* strains, which were validated by PCR and qPCR assays (Figure 1A and Figure S1B). Additionally, the complemented strains were constructed and verified by both PCR and qPCR (Figure 1A and Figure S1B). To elucidate the role of these genes in fungal growth and development, we conducted phenotypic assays on Δ*FpFumA* and Δ*FpFumB* mutants. After 7 days of culturing on PDA medium, the colony diameter of Δ*FpFumB* was significantly reduced by 41% compared to the WT, whereas no significant difference was observed in the colony diameter of the Δ*FpFumA* relative to the WT (Figure 1B). These results suggest that FpFumB plays a crucial role in regulating hyphal vegetative growth. Morphological analysis of conidia revealed that Δ*FpFumA* and Δ*FpFumB* predominantly produced conidia in the 0–20 μm and 20–40 μm ranges, with fewer conidia exceeding 40 μm. In contrast, over 40% of conidia in the WT exceeded 40 μm (Figure 1C), indicating that *FpFUM* genes may regulate spore elongation pathways, such as those involved in cell wall synthesis or microtubule polarization, thereby affecting spore development. Spore production assays revealed a significant reduction in conidia formation at both 24 h and 48 h in the Δ*FpFumA* and Δ*FpFumB* compared to the WT (Figure 1D). In addition, Δ*FpFumA*-C and Δ*FpFumB*-C displayed phenotypes similar to the WT in colony growth, conidiation, and spore morphology (Figure 1B–D). Taken together, these results indicate that the *FpFUM* genes play a key role in regulating vegetative growth, sporulation, and conidial morphological differentiation in *F. proliferatum*.

### 3.2. FpFUM Impacts Environmental Stress Response in Fusarium proliferatum

**Figure 2:**
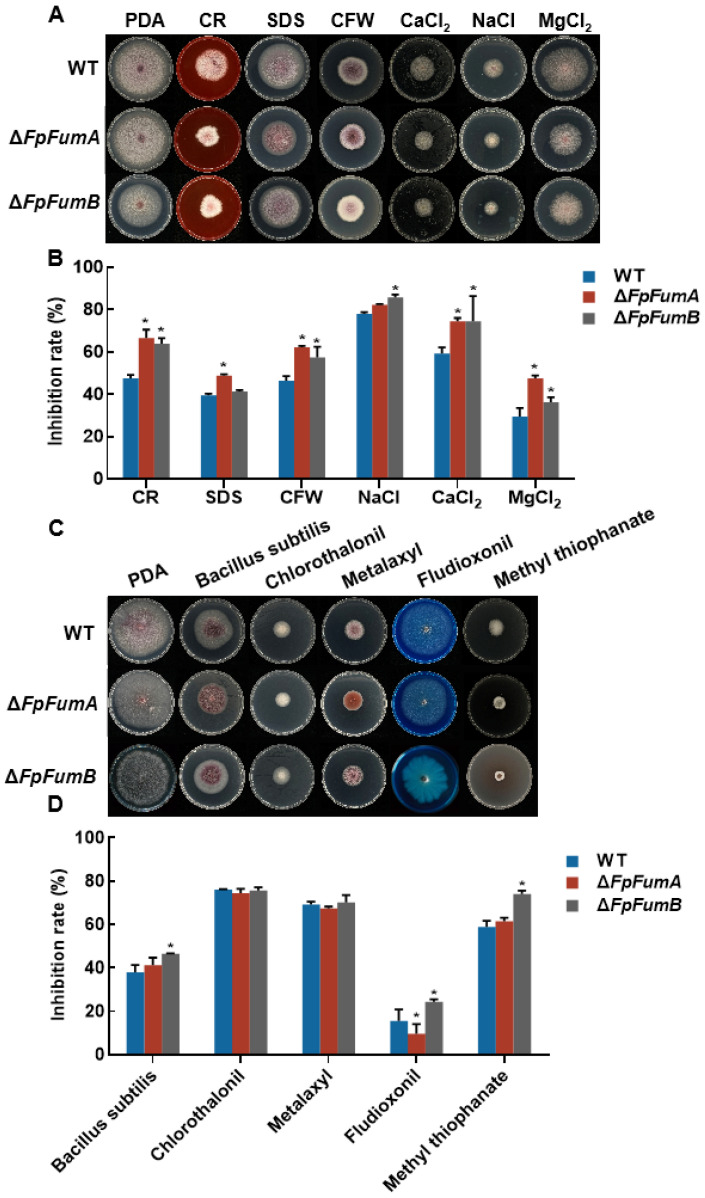
Sensitivity of *Fusarium proliferatum* strains to environmental stressors and antifungal agents. (**A**) Colony morphology of WT, Δ*FpFumA*, and Δ*FpFumB* under control (PDA medium) and stress-inducing conditions: cell wall stressors (0.2% CR, SDS, 0.2% CFW), metal ions (1.4 mol/L CaCl_2_, 1.4 mol/L MgCl_2_), and osmotic stress (2 mol/L NaCl). Representative images display differential growth responses across treatments. (**B**) Inhibition rates (%) of WT, Δ*FpFumA*, and Δ*FpFumB* under stressors CR, SDS, CFW, NaCl, CaCl_2_, and MgCl_2_. (**C**) Colony morphology of WT, Δ*FpFumA*, and Δ*FpFumB* exposed to antifungal agents: *Bacillus subtilis*, chlorothalonil, metalaxyl, fludoxonil, and methyl thiophanate. (**D**) Inhibition rates (%) of strains under antifungal treatments. Data shown are mean + SD. Asterisks indicate a significant difference compared with WT strain (*t*-test, * *p* < 0.05).

Pathogens encounter various environmental stresses during infection. To evaluate the role of *FpFUM* genes in stress responses, the sensitivity of Δ*FpFUM* strains to metal ions, osmotic stress, cell-wall-disrupting agents, and fungicides was assessed. Δ*FpFumA* showed increased sensitivity to cell wall stressors (CR, SDS, CFW) and metal ion stress (CaCl_2_, MgCl_2_), but no significant change in osmotic stress sensitivity (Figure 2A,B). In contrast, Δ*FpFumB* exhibited heightened sensitivity to all tested stressors, suggesting that FpFumB plays a central role in stress tolerance (Figure 2A,B). Fungicide sensitivity assays indicated that Δ*FpFumB* was more susceptible to *Bacillus subtilis* (commercial biological fungicide formulation), fludioxonil, and methyl thiophanate, while Δ*FpFumA* showed increased resistance to fludioxonil (Figure 2C,D). These findings underscore the significance of *FpFUM* genes in facilitating the fungus’s adaptation to environmental stresses, with FpFumB assuming a particularly pivotal role in the modulation of stress resistance.

### 3.3. Role of FpFumB in Regulating Pathogenicity of Fusarium proliferatum

**Figure 3:**
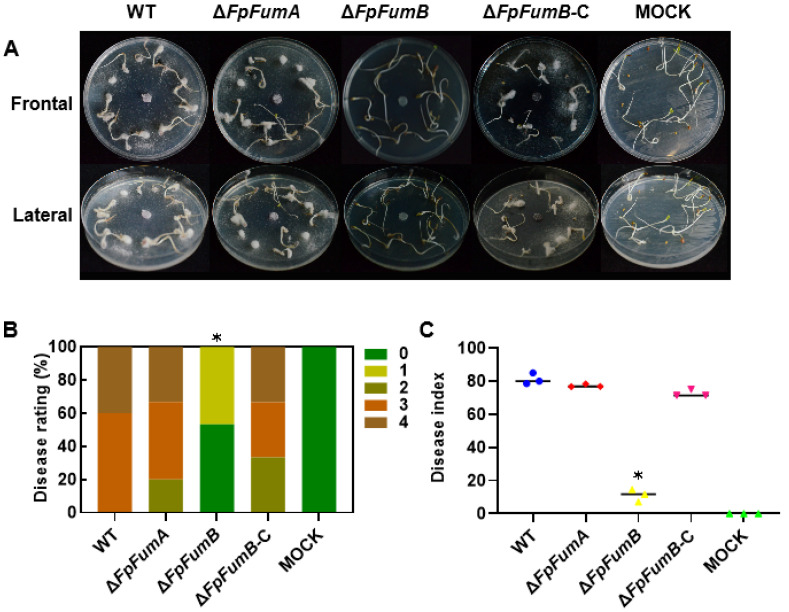
Pathogenicity assay of *Fusarium proliferatum* strains on alfalfa seeds and seedlings. (**A**) Disease phenotypes of seedlings inoculated with WT, Δ*FpFumA*, Δ*FpFumB*, complemented strain *FpFumB*-C, and mock-treated control (MOCK). (**B**) Disease rating percentages across treatment groups, categorized by symptom severity. (**C**) Disease index calculated for each treatment group. Asterisks indicate the level of statistical significance (*t*-test, * *p* < 0.05).

To determine whether *FpFUM* genes are involved in the pathogenic process, we assessed the virulence of the mutants through a *Medicago sativa* seed infection assay. The results showed that after 10 days of inoculation, the disease index of the Δ*FpFumB*-inoculated seeds was 86% lower than that of the WT group, with 53.3% of the seedlings showing no symptoms (0 level) and 46.7% showing slight browning at the root (1 level). In the WT inoculated group, 40% of the seeds failed to germinate and exhibited severe disease (Figure 3A,B). In contrast, the disease index of the Δ*FpFumA*-inoculated seeds did not change significantly. To confirm that the reduced virulence of the Δ*FpFumB* was due to gene knockout, we constructed a complemented strain, Δ*FpFumB*-C, which restored virulence to near WT levels (Figure 3). Additionally, the Δ*FpFumB* mutant was unable to penetrate cellophane membranes, suggesting that its reduced virulence may result from a defect in penetration ability (Figure S2). These findings suggest that FpFumB is the main regulator of the virulence in *F. proliferatum*.

### 3.4. Impact of FpFUM Gene on Mitochondrial Function and Metabolism in Fusarium proliferatum

**Figure 4:**
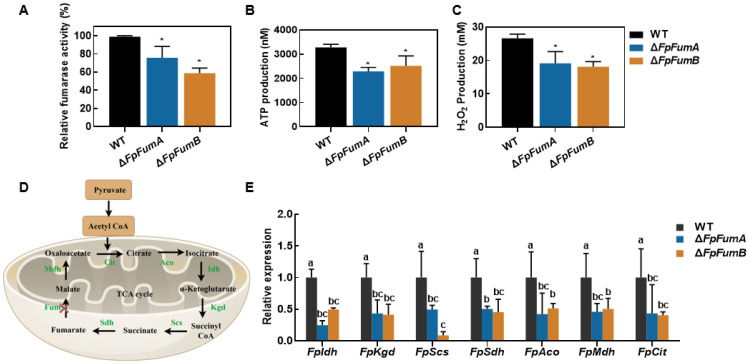
Impact of *FpFumA* and *FpFumB* deletion on mitochondrial metabolism and TCA-cycle-related gene expression in *Fusarium proliferatum*. (**A**) Relative fumarase activity (%) in WT, Δ*FpFumA*, and Δ*FpFumB* strains. (**B**) ATP production and (**C**) H_2_O_2_ production in WT and mutants. (**D**) Schematic diagram of TCA cycle, highlighting key enzymes from pyruvate to fumarate. (**E**) Relative expression levels of TCA-cycle-associated genes (*FpIdh*, *FpKgd*, *FpScs*, *FpSdh*, *FpAco*, *FpMdh*, *FpCit*) in WT, Δ*FpFumA*, and Δ*FpFumB* strains, analyzed by qPCR. Data in panels A–C and E represent mean + SD. Asterisks denote significant differences compared to WT (*t*-test, * *p* < 0.05). Different letters denote between-group comparisons (one-way ANOVA, LSD post hoc test, *p* < 0.05).

Fumarase is a core enzyme in the TCA cycle, and its functional loss can disrupt carbohydrate metabolism, leading to energy supply and mitochondrial metabolic disorders. We found that total fumarase activity decreased by 23.4% in Δ*FpFumA* and by 40.5% in Δ*FpFumB* (Figure 4A). ATP measurements revealed that ATP levels in Δ*FpFumA* and Δ*FpFumB* were reduced by 30.2% and 23.2%, respectively (Figure 4B), indicating impaired energy metabolism. Additionally, hydrogen peroxide (H_2_O_2_) levels in both mutants decreased by 28.1% and 31.8%, respectively (Figure 4C), suggesting mitochondrial oxidative balance disruption. qPCR analysis showed that the expression levels of key TCA cycle enzymes, including isocitrate dehydrogenase (*FpIdh*), α-ketoglutarate dehydrogenase (*FpKgd*), succinyl-CoA synthetase (*FpScs*), succinate dehydrogenase (*FpSdh*), malate dehydrogenase (*FpMdh*), citrate synthase (*FpCit*), and aconitase (*FpAco*), were significantly downregulated in both mutants (Figure 4E). These results provide physiological and biochemical evidence that the *FpFUM* gene deletion disrupts the integrity of the TCA cycle (Figure 4D), leading to electron transport chain dysfunction and metabolic disorders, which in turn affect the normal physiological activities of ***F. proliferatum.***

### 3.5. Exogenous Malate Restores the Phenotypic Defect in ΔFpFumB Mutant

**Figure 5:**
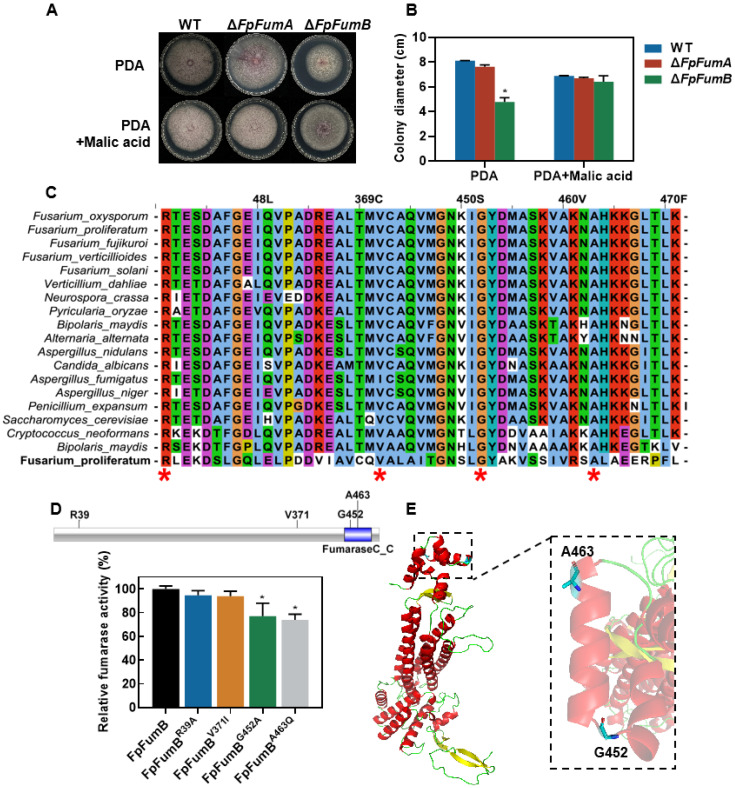
Malate supplementation restores growth defects in Δ*FpFumB* and identification of conserved catalytic residues critical for FpFumB enzymatic activity. (**A**) Colony morphology of WT, Δ*FpFumA*, and Δ*FpFumB* strains on PDA medium and PDA supplemented with malic acid. (**B**) Quantitative comparison of colony diameters for WT, Δ*FpFumA*, and Δ*FpFumB* on PDA and PDA supplemented with malic acid. (**C**) Multiple sequence alignment of conserved regions in fungal fumarase homologs, highlighting conserved residues (*). Different colors represent distinct conserved amino acid sites. (**D**) Relative fumarase activity of purified FpFumB and its site-directed mutants. (**E**) Predicted protein structure of FpFumB, with critical amino acid sites (G452, A463) annotated and highlighted in the 3D model. Values are expressed as mean + SD (B and D). Statistically significant differences relative to WT are marked with asterisks (*t*-test, * *p* < 0.05).

In the TCA cycle, fumarase catalyzes the conversion of fumarate to malate. To determine whether the function of FpFUM is dependent on its metabolic product, we supplemented PDA medium with malate and observed the growth of Δ*FpFumA* and Δ*FpFumB* mutants. The results showed that after adding exogenous malate, the colony growth rate of Δ*FpFumB* mutants was completely restored to WT levels (Figure 5A,B), indicating that FpFumB regulates downstream metabolic networks through catalyzing the fumarate–malate conversion, and the phenotypic defects are related to the blockage of metabolite synthesis.

### 3.6. Identification of Key Amino Acid Sites in FpFumB Protein

To understand the biochemical characteristics of FpFumB, we compared the homologous protein sequences of FpFumB from 19 fungi and selected four highly conserved sites, R39, V371, G452, and A463, as candidate key sites (Figure 5C). Further site-directed mutagenesis of these four sites and protein purification were performed. The fumarase activity of FpFumB mutants (FpFumB^R39A^, FpFumB^V371I,^ FpFumB^G452A^, FpFumB^A463Q^) showed a 23% and 26% decrease in enzyme activity for FpFumB^G452A^ and FpFumB^A463Q^, respectively, while mutations at R39 and V371 did not significantly affect enzyme activity, indicating that G452 and A463 are key amino acid sites of the FpFumB protein (Figure 5D). Domain analysis revealed that G452 and A463 are located within the FumaraseC_C domain (PF10415) (Figure 5D). Structural modeling of the FpFumB protein showed that G452 is located at the junction between two α-helix regions, and A463 is located inside an α-helix region (Figure 5E).

### 3.7. Functional Characterization of Gene Groups Regulated by the FpFumB Gene in Fusarium proliferatum

**Figure 6:**
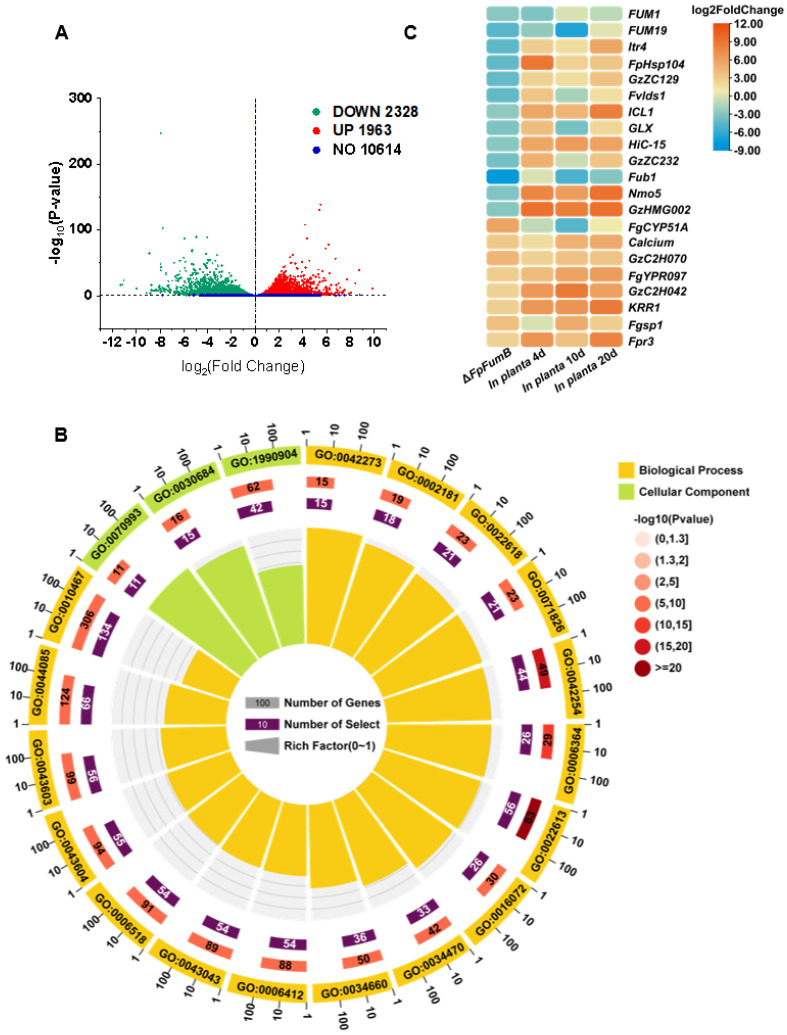
Transcriptome dynamics and functional enrichment reveal FpFumB-mediated metabolic regulation in *Fusarium proliferatum*. (**A**) Volcano plot displaying differentially expressed genes between Δ*FpFumB* and WT strains. (**B**) Circular GO enrichment map illustrating significantly enriched Gene Ontology terms. (**C**) Heatmap of log_2_FoldChange expression values for selected genes in Δ*FpFumB* mutants (compared to WT) and under infection conditions at different time points (*in planta* 2 day, 4 day, 10 day).

To explore the global regulatory network of FpFumB, we conducted RNA-Seq to compare the transcriptomes of WT and Δ*FpFumB* mutants. A total of 4291 differentially expressed genes (DEGs) were identified, with 1963 upregulated and 2328 downregulated (Figure 6A). GO enrichment analysis revealed significant changes in pathways such as ribosomal biogenesis, rRNA processing, ribonucleoprotein complex biogenesis, ribonucleoprotein complex assembly, ribonucleoprotein complex subunit organization, and translation (Figure 6B, Table S2). KEGG pathway analysis further identified that the DEGs were significantly enriched in ribosomal metabolism, such as in the ribosome and ribosome biogenesis in eukaryote pathways, as well as RNA metabolism, including RNA polymerase, RNA degradation, and RNA transport (Table S3). Additionally, a significant enrichment of DEGs was found in the TCA cycle pathway (Table S3). These results suggest a potential link to cellular stress compensation mechanisms, indicating that the loss of FpFumB causes widespread metabolic disturbances. Furthermore, analysis using the PHI database identified 20 potential pathogenicity-related genes among the DEGs, with expression changes greater than twofold in Δ*FpFumB*, showing differential expression under host-induced conditions (Figure 6C, Table S4). These genes, including integral membrane protein *Fgsg03009*, glucoside transport protein *FGSG_0461*, ATPase *FgPMA2,* fumonisin cluster *FUM19*, and various transcription factors (*GzZC297*, *GzZC045*, *GzZC180*, *GzZC129*, *GzZC067)*, are involved in transcription regulation, toxin synthesis, and signal transduction (Figure 6C, Table S4). The significant changes in their expression may contribute to the pathogenicity of *F. proliferatum* caused by *FpFumB* deletion.

### 3.8. FpFumB Regulates DNA Damage Repair Function

**Figure 7:**
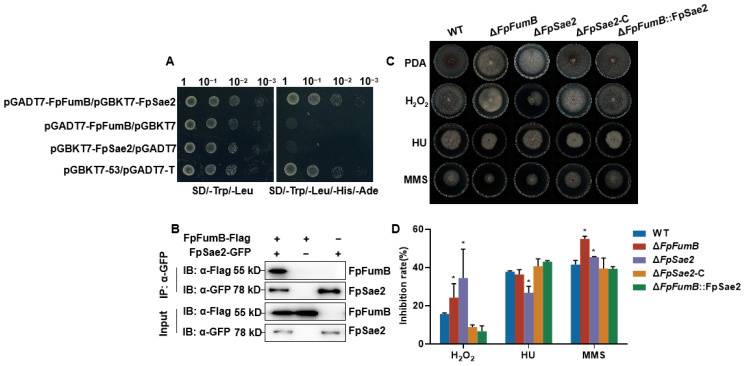
FpFumB interacts with FpSae2 to regulate DNA damage repair in *Fusarium proliferatum.* (**A**) Interaction between FpFumB and FpSae2 in Y2H assays. (**B**) Interaction between FpFumB and FpSae2 in Co-IP assays. (**C**) Colony morphology of WT, Δ*FpFumB*, Δ*FpSae2*, complemented strain (Δ*FpSae2*-C), and overexpression strain (Δ*FpFumB*::FpSae2) on PDA medium under 2 mmol/L H_2_O_2_, 10 mmol/L hydroxyurea (HU), or 30 mmol/L methyl methanesulfonate (MMS) treatments. (**D**) Inhibition rates (%) of strains under H_2_O_2_, HU, and MMS treatments. Data in D are expressed as mean + SD. Statistically significant differences relative to WT are marked with asterisks (*t*-test, * *p* < 0.05).

Fumarase is involved in the DNA damage repair process. To investigate the role of FpFumB in DNA repair, we examined its interaction with the DNA repair protein FpSae2 (FPRO_07713, XM_031234200). Y2H assays showed that co-transformation of pGADT7-FpFumB and pGBKT7-FpSae2 led to yeast growth on QDO, whereas single transformations did not result in growth (Figure 7A). Co-IP assays further confirmed that FpFumB-FLAG interacts with FpSae2-GFP in *Fusarium proliferatum* (Figure 7B, Figure S3A). Subsequently, Δ*FpSae2* mutants, complemented strains Δ*FpSae2*-C, and overexpression strains Δ*FpFumB*::FpSae2 were constructed (Figure S3B). The sensitivity of these strains to DNA-damaging agents was tested. Both Δ*FpFumB* and Δ*FpSae2* showed increased sensitivity to H_2_O_2_ and MMS treatments, while Δ*FpSae2* exhibited increased sensitivity to HU. However, sensitivity to these three agents was restored to WT levels in the Δ*FpSae2*-C and Δ*FpFumB*::FpSae2 strains (Figure 7C,D). These results suggest that FpFumB participates in regulating DNA damage repair through its interaction with the DNA repair protein FpSae2.

### 3.9. Role of FpSae2 in Regulating Growth, Sporulation, and Pathogenicity of Fusarium proliferatum

**Figure 8:**
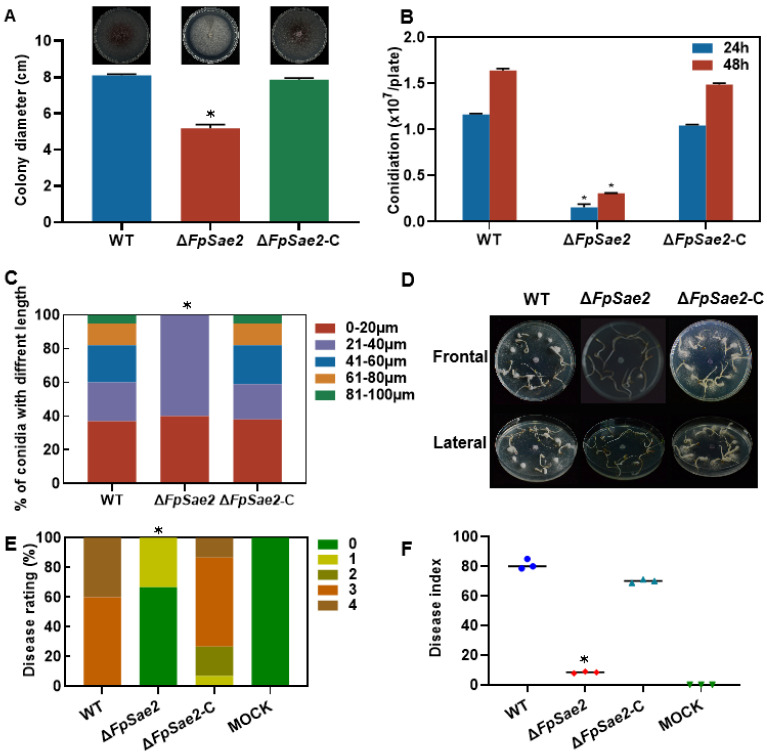
Phenotypic and pathogenicity analysis of Δ*FpSae2* mutant. (**A**) Colony diameter and morphology of WT, Δ*FpSae2*, and complemented strain Δ*FpSae2*-C on PDA medium. (**B**) Conidiation levels of WT, Δ*FpSae2*, and Δ*FpSae2*-C at 24 h and 48 h post-inoculation. (**C**) Percentage distribution of conidial lengths in WT, Δ*FpSae2*, and Δ*FpSae2*-C. (**D**) Disease phenotypes and (**E**) disease rating percentages categorized by symptom severity in alfalfa seedlings inoculated with WT, Δ*FpSae2*, Δ*FpSae2*-C, and mock control. (**F**) Disease index of inoculated seedlings. Data in A and B are expressed as mean + SD. Statistically significant differences relative to WT are marked with asterisks (*t*-test, * *p* < 0.05).

To clarify the role of FpSae2, we generated gene knockout mutant Δ*FpSae2* and complemented strain Δ*FpSae2*-C, and verified the transformants by PCR (Figure S3C,D). Phenotypic analysis showed that the growth rate of Δ*FpSae2* decreased by 36% compared to the WT (Figure 8A). Spore production was also significantly reduced, with 87% and 82% decreases at 24 h and 48 h, respectively (Figure 8B). Spore morphology analysis revealed that the conidia length in Δ*FpSae2* was restricted to 0–40 μm, while over 40% of the spores in WT and Δ*FpSae2*-C strains exceeded 40 μm (Figure 8C). Pathogenicity assays indicated that the disease index of alfalfa seeds and seedlings inoculated with Δ*FpSae2* was reduced by 85% compared to the WT, with 66.7% of the inoculated seedlings showing no symptoms (grade 0). The virulence of the Δ*FpSae2*-C strain was restored to WT levels. These results demonstrate that FpSae2 regulates the growth, sporulation, spore morphology, and pathogenicity of *F. proliferatum*.

## 4. Discussion

As a pivotal enzyme catalyzing the reversible hydration of fumarate to malate, fumarase occupies a central control point in the TCA cycle [13]. In plant pathogenic fungi, the disruption of TCA cycle enzymes frequently results in compromised development and pathogenicity. In *Fusarium oxysporum f.* sp. *niveum*, deletion of malate dehydrogenase (*FonMdh2*) caused severe defects in growth, sporulation, and a complete loss of virulence [38]. Likewise, knocking out *FgSDHC1*, encoding a succinate dehydrogenase subunit in *Fusarium graminearum*, impaired mycelial growth and reduced virulence [39]. However, despite its recognition as a potential target of dithiocarbamate fungicides, the biological function of fumarase in phytopathogenic fungi has remained largely uncharacterized. Our study elucidates the role of fumarase in hyphal growth, stress response, and pathogenicity in *F. proliferatum* (Figure 1, Figure 2 and Figure 3), laying a theoretical foundation for targeting key TCA cycle enzymes in fungicide development and resistance mechanism exploration.

Beyond metabolic roles, fumarase family members facilitate DNA repair: yeast fumarase recruits Sae2 to double-strand breaks during homologous recombination [13], and bacterial class-I fumarases interact with RecN to bolster oxidative damage repair [22]. We show that FpFumB binds FpSae2 (Figure 7A,B) and that Δ*FpSae2* mimics Δ*FpFumB* in sensitivity to H_2_O_2_, HU, and MMS as well as in virulence loss (Figure 7C,D and Figure 8D–F). Moreover, overexpression of FpSae2 rescues the DNA repair and pathogenicity defects of Δ*FpFumB* (Figure 7C,D). These findings, mirroring the yeast model, reveal a conserved mechanism in which a metabolic enzyme directly scaffolds a repair factor to maintain genome integrity and pathogenic potential in phytopathogenic fungi.

Site-directed mutagenesis and structural modeling revealed that G452 and A463, two residues embedded within the conserved FumaraseC_C domain, are critical for FpFumB catalytic activity, as their mutation led to a 23–26% reduction in enzyme function (Figure 5D). G452 is located at the junction between two α-helices, a region known for its conformational flexibility, while A463 resides within a helical segment, likely contributing to the stabilization of the enzyme tertiary structure (Figure 5E). The identification of these critical sites strengthens the case for fumarase as a potential target for antifungal strategies. Importantly, these findings provide theoretical support for the development of antifungal agents targeting enzymes within the TCA cycle, as several fungicides are known to exert their action by interfering with energy metabolism [40]. Enzymes involved in glycolysis, fatty acid β-oxidation, acetyl-CoA synthesis, the TCA cycle, the electron transport chain, and alternative oxidase pathways have all been identified as fungicide targets [41]. For instance, dithiocarbamate fungicides disrupt respiration by chelating iron cofactors essential for aconitase activity, leading to TCA cycle arrest [42]. Similarly, SDHI (succinate dehydrogenase inhibitor) fungicides target the succinate dehydrogenase complex, effectively blocking mitochondrial electron transport [43,44].

## 5. Conclusions

In summary, this study demonstrates that FpFumB is essential for basic biological processes and virulence in *Fusarium proliferatum*. FpFumB not only functions in the TCA cycle and energy metabolism but also contributes to DNA damage repair by interacting with the repair protein FpSae2. Loss of *FpFumB* leads to impaired mitochondrial function, reduced growth and stress tolerance, and significantly weakened pathogenicity. These results highlight the connection between primary metabolism and the DNA repair pathway, providing a theoretical basis for targeting fumarase as a potential antifungal strategy.

## Data Availability

The original contributions presented in this study are included in the article/Supplementary Material. Further inquiries can be directed to the corresponding author.

## Supplementary Materials

The following supporting information can be downloaded at: https://www.mdpi.com/article/10.3390/microorganisms13061433/s1, Figure S1: Validation of *FpFum* genes knockout mutants and complemented strains; Figure S2: Cellophane membrane penetration assay; Figure S3: Molecular characterization of strains related to *FpSae2* gene manipulation; Table S1: Primers used in this study; Table S2: GO number and its corresponding function description for differentially expressed genes in Δ*FpFumB*; Table S3: KEGG pathway enrichment entries for differentially expressed genes in Δ*FpFumB*; Table S4: PHI database identified 20 potential pathogenicity-related genes among the differentially expressed genes in Δ*FpFumB*; Table S5: Strains used in this study and their relevant genotypes.

## Abbreviations

The following abbreviations are used in this manuscript:

CWDEs	cell-wall-degrading enzymes
ROS	reactive oxygen species
VOCs	volatile organic compounds
TCA	tricarboxylic acid
WT	wild-type
PDA	potato dextrose agar
PDB	potato dextrose broth
CMC	carboxymethyl cellulose
CR	Congo Red
CFW	Calcofluor White
SDS	sodium dodecyl sulfate
HU	hydroxyurea
MMS	methyl methanesulfonate
qPCR	quantitative PCR
WA	water agar
GO	Gene Ontology
KEGG	Kyoto Encyclopedia of Genes and Genomes
RLU	relative light units
IPTG	isopropyl β-D-1-thiogalactopyranoside
DEGs	differentially expressed genes
H_2_O_2_	hydrogen peroxide
