# Supplementary material for: FpFumB Is Required for Basic Biological Processes and Virulence in Fusarium proliferatum by Modulating DNA Repair Through Interaction with FpSae2
Source: Microorganisms. 2025 Jun 19;13(6):1433. doi: 10.3390/microorganisms13061433 (PMC12196060; doi:10.3390/microorganisms13061433)
Supplement: Supplementary file 1 [file microorganisms-13-01433-s001.zip › microorganisms-3638142-supplementary.pdf]

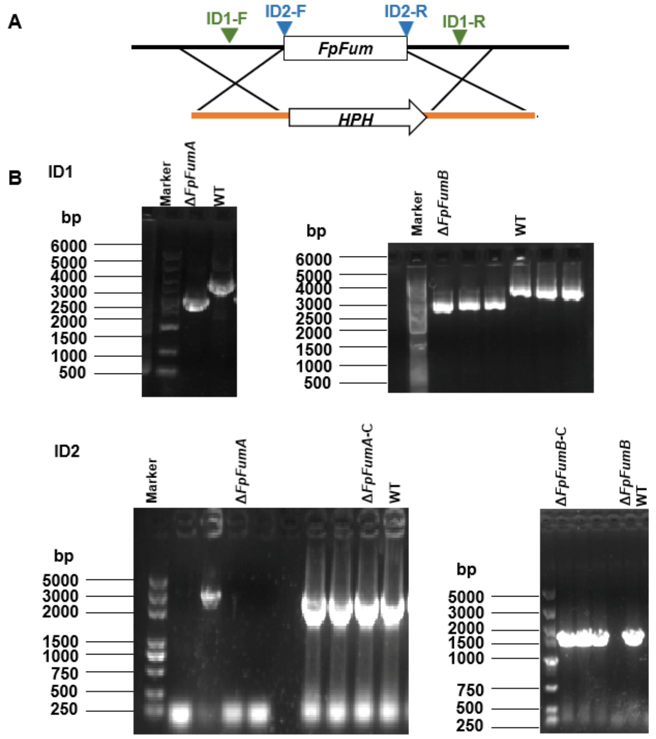

Figure S1 Validation of *FpFum* genes knockout mutants and complemented strains. (A) Schematic diagram of *FpFum*-targeted knockout strategy. ID1 and ID2 are the primers used in PCR identification. (B) PCR identification of the *FpFum*-knockout mutants and complemented strains.

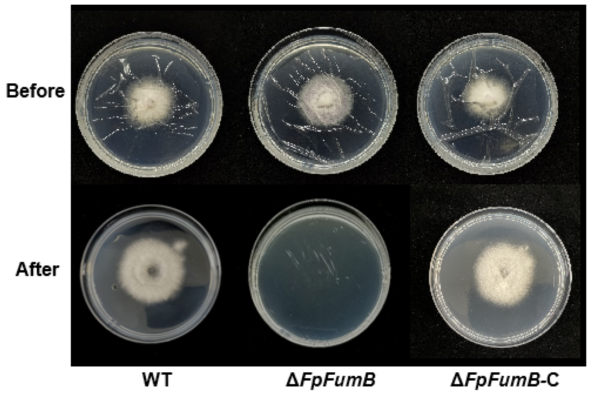

Figure S2 Cellophane membrane penetration assay. Hyphal blocks from WT, Δ*FpFumB*, and Δ*FpFumB*-C were inoculated on cellophane membranes overlaid on PDA medium for 3 days (Before). The cellophane membrane were removed, and the resulting plates were incubated for 3 additional days (After).

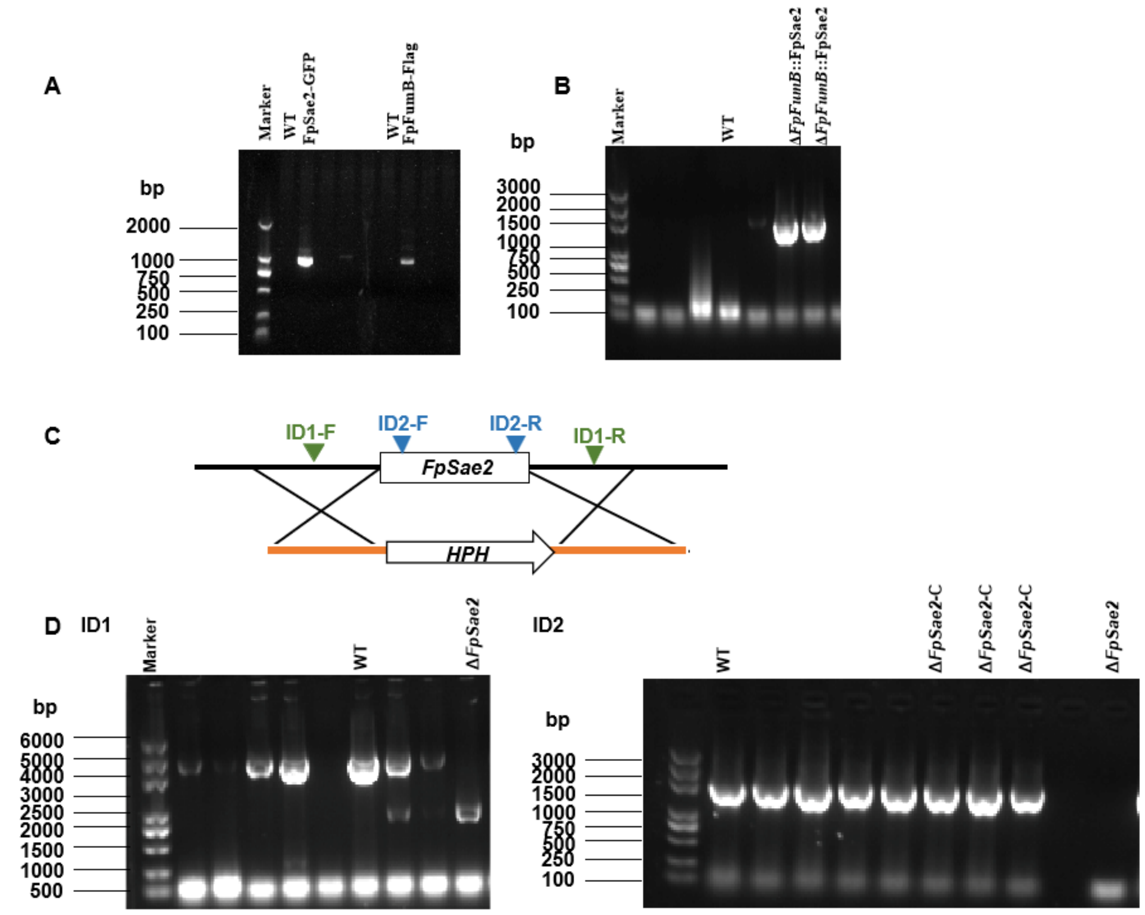

Figure S3 Molecular characterization of strains related to *FpSae2* gene manipulation. (A) PCR verification of the Co-Immunoprecipitation strain, confirming the overexpression of FpSae2-GFP and FpFumB-Flag in the WT background. (B) PCR verification confirming the overexpression of FpSae2 in the Δ*FpFumB*::FpSae2 strain. (C) Schematic representation of the *FpSae2* gene disruption strategy. ID1 and ID2 indicate the primer pairs used for PCR screening. (D) PCR verification of *FpSae2* deletion mutants and their corresponding complemented strains.

Table S1 Primers used in this study

Primer name	Sequence（5'-3'）
HPH-F	GGAGGTCAACACATCAATGCCTATT
HPH-R	CTACTCTATTCCTTTGCCCT
FpFumA-5UTR-F	CTGAAGCACCCGTAAACAA
FpFumA-5UTR-R	CAAAATAGGCATTGATGTGTTGACCTCCGAAAGGGCGAGATGAAGAG
FpFumA-3UTR-F	CGTCCGAGGGCAAAGGAATAGAGTAGAAGATCCACAGGCTTCAAGA
FpFumA-3UTR-R	ATCTGCACCGAGGTGGCA
FpFumB-5UTR-F	GAAATGGCTGAAGCGGGTA
FpFumB-5UTR-R	CAAAATAGGCATTGATGTGTTGACCTCCCCGAAAGAAAGAAGAAAGGAAC
FpFumB-3UTR-F	CGTCCGAGGGCAAAGGAATAGAGTAGTACTCGAACCTGCAAACCA
FpFumB-3UTR-R	GTCTCGTCAGCCGTAACCT
FpSae2-5UTR-F	ACGGGCAATATCTGGTTC
FpSae2-5UTR-R	CAAAATAGGCATTGATGTGTTGACCTCCTTCTGTTGACTCGTCTTCC
FpSae2-3UTR-F	CGTCCGAGGGCAAAGGAATAGAGTAGGGAGGAGGCAACTCAATG
FpSae2-3UTR-R	CTGGCTGAGGAAATCATAGA
FpFumB-R39-F	GACAAAGGCGCTCGAAAAAGACAGTCTCGGTCA
FpFumB-R39-R	TTTCGAGCGCCTTTGTCTCGATGGTAACGGTCA
FpFumB-V371-F	TGTCAAATCGCATTGGCCATCACTGGAAATGA
FpFumB-V371-R	GCCAATGCGATTTGACAAACCGCAATGGGAAT
FpFumB-G452-F	AAGCCTTGCGTATGCCAAAGTGTCAAGCATCG
FpFumB-G452-R	TGGCATACGCAAGGCTTGGGACAAGAGCTGTT
FpFumB-A463-F	TGAGATCGCAACTGGCTGAGGAGCGCCCGTTT
FpFumB-A463-R	AGCCAGTTGCGATCTCACGATGCTTGACACTT
FpFumA-BD-F	ATGGCCATGGAGGCCGAATTCATGCTTCGAACCGTCACG
FpFumA-BD-R	CCGCTGCAGGTCGACGGATCCTCCCTTGTAGGGGCTGG
FpFumA-His-F	GCCATGGCTGATATCGGATCCATGCTTCGAACCGTCACG
FpFumA-His-R	GTGGTGGTGGTGGTGCTCGAGTCCCTTGTAGGGGCTGG

FpFumB-AD-F	GCCATGGAGGCCAGTGAATTCATGACGGTCACTCAAATCAT
FpFumB-AD-R	CAGCTCGAGCTCGATGGATCCCTCGATGAAAACCGAACGC
FpFumB-Flag-F	CTATAGGGCGAATTGGGTACTCAAATTGGTTGAAATGGCTGAAGCGGGTA
FpFumB-Flag-R	CTTTATAATCACCGTCATGGTCTTTGTAGTCCTCGATGAAAACCGAACGC
FpFumB-His-F	GCCATGGCTGATATCGGATCCATGACGGTCACTCAAATCAT
FpFumB-His-R	GTGGTGGTGGTGGTGCTCGAGCTCGATGAAAACCGAACGC
FpSae2-AD-F	GCCATGGAGGCCAGTGAATTCATGACGAGTTGGCTTGCTTCTGG
FpSae2-AD-R	CAGCTCGAGCTCGATGGATCCCTCATCGCGGAACAGCCATCTAC
FpSae2-BD-F	ATGGCCATGGAGGCCGAATTCATGACGAGTTGGCTTGCTTCTGG
FpSae2-BD-R	CCGCTGCAGGTCGACGGATCCCTCATCGCGGAACAGCCATCTAC
FpSae2-GFP-F	TTTCGTAGGAACCCAATCTTCAAAATGACGAGTTGGCTTGCTTCTGG
FpSae2-GFP-R	CACCACCCCGGTGAACAGCTCCTCGCCCTTGCTCACCTCATCGCGGAACAGCCATCTAC
FpSae2-QC-F	CTACGCTGAAGGACAAACG
FpSae2-QC-R	GCGTCTACTCTTCGGCTTCG
FpSae2-MD-F	ATGACGAGTTGGCTTGCTTCTGG
FpSae2-MD-R	CTCATCGCGGAACAGCCATCTAC
G418-F	GGAGGTCAACACATCAATGCT
G418-R	TCAGAAGAACTCGTCAAGAAG
FpFumB-Flag-ID-F	TCGTGCCATGGAGGGTGTTT
FpFumB-Flag-ID-R	AATGTTGAGTGGAATGATTTA
FpSae2-GFP-ID-F	ATTGAGCGTAAATCGAAGG
FpSae2-GFP-ID-R	GTCAGCTTGCCGTAGGTGGCA

Table S2 GO number and its corresponding function description for differentially expressed genes in Δ*FpFumB*

ID	Classification	Description
GO:0022613	Biological Process	ribonucleoprotein complex biogenesis
GO:0042254	Biological Process	ribosome biogenesis
GO:0006364	Biological Process	rRNA processing
GO:0034470	Biological Process	ncRNA processing
GO:0016072	Biological Process	rRNA metabolic process
GO:1990904	Cellular Component	ribonucleoprotein complex
GO:0022618	Biological Process	ribonucleoprotein complex assembly
GO:0071826	Biological Process	ribonucleoprotein complex subunit organization
GO:0034660	Biological Process	ncRNA metabolic process
GO:0006412	Biological Process	translation
GO:0043043	Biological Process	peptide biosynthetic process
GO:0002181	Biological Process	cytoplasmic translation
GO:0006518	Biological Process	peptide metabolic process
GO:0042273	Biological Process	ribosomal large subunit biogenesis
GO:0043604	Biological Process	amide biosynthetic process
GO:0043603	Biological Process	cellular amide metabolic process
GO:0044085	Biological Process	cellular component biogenesis
GO:0030684	Cellular Component	preribosome
GO:0010467	Biological Process	gene expression
GO:0070993	Cellular Component	translation preinitiation complex

Table S3 KEGG pathway enrichment entries for differentially expressed genes in Δ*FpFumB*

ID	Description	Test	TestAll	Pvalue
ko03010	Ribosome	69	690	5.75E-19
ko03008	Ribosome biogenesis in eukaryotes	48	690	7.96E-15
ko03020	RNA polymerase	19	690	5.38E-07
ko04623	Cytosolic DNA-sensing pathway	10	690	0.000159
ko00100	Steroid biosynthesis	16	690	0.002987
ko03040	Spliceosome	37	690	0.003142
ko04213	Longevity regulating pathway - multiple species	14	690	0.011058
ko00640	Propanoate metabolism	12	690	0.017277
ko00410	beta-Alanine metabolism	19	690	0.019691
ko00232	Caffeine metabolism	3	690	0.025075
ko03018	RNA degradation	20	690	0.027262
ko04973	Carbohydrate digestion and absorption	5	690	0.025797
ko00630	Glyoxylate and dicarboxylate metabolism	17	690	0.039232
ko00710	Carbon fixation in photosynthetic organisms	10	690	0.057602
ko00903	Limonene and pinene degradation	4	690	0.064801
ko00981	Insect hormone biosynthesis	4	690	0.064801
ko03013	RNA transport	19	690	0.065753
ko00010	Glycolysis / Gluconeogenesis	20	690	0.203031
ko00020	Citrate cycle (TCA cycle)	8	690	0.605134
ko00030	Pentose phosphate pathway	9	690	0.439798

Table S4 PHI database identified 20 potential pathogenicity-related genes among the differentially expressed genes in Δ*FpFumB*

PHI ID	Gene Name	Gene ID	Product Description
PHI:5834	Fgsg03009	FPRO_15630	related to integral membrane protein
PHI:1094	FGSG_04610	FPRO_14911	related to alpha-glucoside transport protein
PHI:1982	GzZC297	FPRO_14240	related to STB5-transcription factor
PHI:1730	GzZC045	FPRO_14182	related to transcriptional activator Mut3p
PHI:9268	FUM19	FPRO_13580	fumonisin cluster-ABC transporter
PHI:9425	Itr4	FPRO_13503	related to myo-inositol transport protein ITR1
PHI:1865	GzZC180	FPRO_10147	related to PPR1-transcription factor regulating pyrimidine pathway
PHI:1814	GzZC129	FPRO_10090	related to cutinase transcription factor 1 beta
PHI:123070	FgPMA2	FPRO_09311	probable PMA1-H+-transporting P-type ATPase
PHI:1269	FGSG_02488	FPRO_08399	related to dis1-suppressing protein kinase dsk1
PHI:305	ICL1	FPRO_08144	probable isocitrate lyase (acu-3)
PHI:1752	GzZC067	FPRO_08120	probable transcription activator protein acu-15
PHI:5393	GLX	FPRO_07068	related to glyoxal oxidase precursor
PHI:11078	Fpnuc1	FPRO_05076	probable nuclease NUC1 precursor, mitochondrial
PHI:6076	RPS41	FPRO_09714	probable ribosomal protein S4.e, cytosolic
PHI:1406	GzC2H070	FPRO_09222	related to Found in Mitochondrial Proteome
PHI:1414	GzC2H081	FPRO_06364	related to dnaJ-like proteins
PHI:5232	MoARG1	FPRO_05993	probable arginosuccinate synthetase
PHI:11628	FoATG12	FPRO_03420	probable APG12-component of the autophagic system
PHI:2548	KRR1	FPRO_02557	probable KRR1 protein, required for 40S ribosome biogenesis

Table S5 Strains used in this study and their relevant genotypes.

Organism	Strain Name	Relevant Genotype / Purpose	Selection Marker
Fusarium proliferatum	HM19-1-1	Wild-Type (WT) strain	-
	Δ*FpFumA*	FpFumA knockout in WT background	Hygromycin
	Δ*FpFumB*	FpFumB knockout in WT background	Hygromycin
	Δ*FpFumA*-C	Δ*FpFumA* with *FpFumA* gene reintroduced via pYF11 vector	G418
	Δ*FpFumB*-C	Δ*FpFumB* with *FpFumB* gene reintroduced via pYF11 vector	G418
	Δ*FpSae2*	FpSae2 knockout in WT background	Hygromycin
	Δ*FpSae2*-C	Complemented strain of ΔFpSae2	G418
	Co-IP Strain	WT co-expressing FpSae2-GFP (from pYF11) and FpFumB-Flag (from pHZ126)	G418 and Hygromycin
	Δ*FpFumB*::FpSae2	FpSae2-GFP overexpressed in the ΔFpFumB mutant background via pYF11	G418 and Hygromycin
Saccharomyces cerevisiae	Y2H Gold (pGADT7-FpFumB / pGBKT7-FpSae2)	Y2H: Test for FpFumB and FpSae2 interaction	SD/-Trp/-Leu/-His/-Ade
	Y2H Gold (pGADT7-FpFumB / pGBKT7)	Y2H: Negative control	SD/-Trp/-Leu
	Y2H Gold (pGADT7 / pGBKT7-FpSae2)	Y2H: Negative control	SD/-Trp/-Leu
	Y2H Gold (pGADT7-T / pGBKT7-53)	Y2H: Positive control	SD/-Trp/-Leu/-His/-Ade

Escherichia coli	BL21 (pET32a-FpFumB)	Expression of His-tagged wild-type FpFumB protein	Carbenicillin
	BL21 (pET32a-FpFumB R39A)	Expression of His-tagged mutant FpFumB protein	Carbenicillin
	BL21 (pET32a-FpFumB V371I)	Expression of His-tagged mutant FpFumB protein	Carbenicillin
	BL21 (pET32a-FpFumB G452A)	Expression of His-tagged mutant FpFumB protein	Carbenicillin
	BL21 (pET32a-FpFumB A463Q)	Expression of His-tagged mutant FpFumB protein	Carbenicillin
